# Supplementary material for: FoxP2 isoforms delineate spatiotemporal transcriptional networks for vocal learning in the zebra finch
Source: eLife. 2018 Jan 23;7:e30649. doi: 10.7554/eLife.30649 (PMC5826274; doi:10.7554/eLife.30649)
Supplement: Figure 1—source data 2. [file elife-30649-fig1-data2.zip › Figure1_Source Data 2.pdf]

pcDNA4/HisMax-tFoxP2

CMV promoter

SpeI

1 ATGTACGGGC CAGATATACG CGTTGACATT GATTATTGAC TAGTTATTAA TAGTAATCAA TTACGGGGTC ATTAGTTCAT AGCCCATATA TGGAGTTCCG

CMV promoter

101 CGTTACATAA CTTACGGTAA ATGGCCCGCC TGGCTGACCG CCCAACGACC CCCGCCCATT GACGTCAATA ATGACGTATG TTCCCATAGT AACGCCAATA

CMV promoter

201 GGGACTTTCC ATTGACGTCA ATGGGTGGAG TATTTACGGT AAACTGCCCA CTTGGCAGTA CATCAAGTGT ATCATATGCC AAGTACGCCC CCTATTGACG

CMV promoter

NcoI

301 TCAATGACGG TAAATGGCCC GCCTGGCATT ATGCCCAGTA CATGACCTTA TGGGACTTTC CTACTTGGCA GTACATCTAC GTATTAGTCA TCGCTATTAC

CMV promoter

NcoI

401 CATGGTGATG CGGTTTTGGC AGTACATCAA TGGGCGTGGA TAGCGGTTTG ACTCACGGGG ATTTCCAAGT CTCCACCCCA TTGACGTCAA TGGGAGTTTG

CMV promoter

501 TTTTGGCACC AAAATCAACG GGACTTTCCA AAATGTCGTA ACAACTCCGC CCCATTGACG CAAATGGGCG GTAGGCGTGT ACGGTGGGAG GTCTATATAA

CMV promoter PmeI

SacI NheI

601 GCAGAGCTCT CTGGCTAACT AGAGAACCCA CTGCTTACTG GCTTATCGAA ATTAATACGA CTCACTATAG GGAGACCCAA GCTGGCTAGC GTTTAAACTT

SmaI

XmaI

HindIII AvaI

701 AAGCTTAGCG CAGAGGCTTG GGGCAGCCGA GCGGCAGCCA GGCCCCGGCC CGGGCCTCGG TTCCAGAAGG GAGAGGAGCC CGCCAAGGCG CGCAAGAGAG

initiation ATG 6xHis

NcoI

801 CGGGCTGCCT CGCAGTCCGA GCCGGAGAGG GAGCGCGAGC CGCGCCGGCC CCGGACGGCC TCCGAAACCA TGGGGGGTTC TCATCATCAT CATCATCATG

Xpress(tm) epitope 3'-T overhang

NheI EK recognition site tFoxP2

+3 Met Met Gln Glu Ser Ala Thr Glu Thr

901 GTATGGCTAG CATGACTGGT GGACAGCAAA TGGGTCGGGA TCTGTACGAC GATGACGATA AGGTACAGGC CCTTATGATG CAGGAATCTG CGACAGAGAC

tFoxP2

+3 Thr Ile Ser Asn Ser Ser Met Asn Gln Asn Gly Met Ser Thr Leu Ser Ser Gln Leu Asp Ala Gly Ser Arg Asp Gly Arg Ser Ser Gly Asp Thr Ser Thr

1001 AATAAGCAAC AGTTCAATGA ATCAAAATGG AATGAGCACT CTAAGCAGCC AATTAGATGC TGGCAGCAGA GATGGAAGAT CAAGTGGTGA CACGAGCACT

tFoxP2
PvuII
+3 Glu Val Ser Thr Val Glu Leu Leu His Leu Gln Gln Gln Gln Ala Leu Gln Ala Ala Arg Gln Leu Leu Leu Gln Gln Gln Thr Ser Gly Leu Lys Ser Pro·
1101 GAAGTAAGCA CAGTAGAACT GCTGCATCTG CAACAACAGC AGGCACTCCA GGCAGCAAGG CAGCTGCTTT TACAGCAACA GACAAGTGGA CTGAAATCTC

tFoxP2
PstI MscI
+3 ·Pro Lys Gly Ser Glu Lys Gln Arg Pro Leu Gln Val Pro Val Ser Val Ala Met Met Thr Pro Gln Val Ile Thr Pro Gln Gln Met Gln Gln Ile Leu Gln
1201 CTAAGGGCAG TGAGAAACAG AGACCGCTGC AGGTGCCTGT GTCAGTGGCC ATGATGACTC CCCAGGTGAT CACCCCTCAG CAAATGCAGC AGATTCTCCA

tFoxP2
+3 ·Glr Gln Gln Val Leu Ser Pro Gln Gln Leu Gln Ala Leu Leu Gln Gln Gln Gln Ala Val Met Leu Gln Gln Gln Gln Leu Gln Glu Phe Tyr Lys Lys Gln
1301 GCAGCAAGTG TTGTCTCCGC AGCAGCTTCA AGCACTTCTC CAGCAGCAGC AAGCAGTTAT GTTGCAGCAG CAACAACTAC AAGAGTTTTA CAAGAAACAG

tFoxP2
+3 Gln Glu Gln Leu His Leu Gln Leu Leu Gln Gln Gln Gln Gln Gln Gln Gln Gln Gln Gln Gln Gln Gln Gln Gln Gln Gln Gln Gln Gln Gln Gln Gln
1401 CAAGAGCAGT TACATCTTCA GCTTTTGCAG CAGCAGCAGC AACAGCAGCA GCAACAGCAA CAACAGCAGC AACAACAGCA ACAACAGCAG CAGCAGCAAC

tFoxP2
+3 ·Glr Gln Gln Gln Gln Gln Gln Gln Gln Gln Gln Gln Gln Gln His Pro Gly Lys Gln Ala Lys Glu Gln Gln Gln Gln Gln Gln Gln Gln Leu Ala Ala Gln
1501 AACAACAACA GCAGCAGCAG CAACAGCAAC AGCAGCAACA GCATCCAGGA AAGCAAGCAA AAGAGCAACA GCAGCAGCAG CAGCAGCAGT TGGCAGCCCA

tFoxP2
MscI
+3 ·Glr Gln Leu Val Phe Gln Gln Gln Leu Leu Gln Met Gln Gln Leu Gln Gln Gln Gln His Leu Leu Asn Leu Gln Arg Gln Gly Leu Ile Ser Ile Pro Pro
1601 GCAGCTTGTC TTCCAGCAGC AGCTCCTCCA GATGCAACAA CTTCAGCAGC AGCAACATCT GCTGAACCTT CAGCGTCAGG GACTCATTTC CATCCCACCT

tFoxP2
MscI
+3 Gly Gln Ser Ala Leu Pro Val Gln Ser Leu Pro Gln Ala Gly Leu Ser Pro Ala Glu Ile Gln Gln Leu Trp Lys Glu Val Thr Gly Val His Ser Met Glu
1701 GGCCAATCTG CTCTTCCTGT CCAGTCTCTG CCACAAGCTG GCTTAAGTCC TGCTGAGATT CAGCAGTTAT GGAAAGAAGT GACTGGAGTT CACAGTATGG

tFoxP2
+3 ·Glu Asp Asn Gly Ile Lys His Gly Gly Leu Asp Leu Thr Thr Asn Asn Ser Ser Ser Thr Thr Ser Ser Thr Thr Ser Lys Ala Ser Pro Pro Ile Thr His·
1801 AAGACAATGG CATTAAACAT GGAGGGCTAG ACCTCACTAC TAACAATTCC TCCTCTACTA CCTCCTCCAC CACTTCCAAA GCATCACCAC CAATAACTCA

tFoxP2
+3 ·His His Ser Ile Val Asn Gly Gln Ser Ser Val Leu Asn Ala Arg Arg Asp Ser Ser Ser His Glu Glu Thr Gly Ala Ser His Thr Leu Tyr Gly His Gly
1901 TCATTCCATA GTGAATGGAC AGTCTTCAGT TCTAAATGCA AGGCGAGACA GCTCGTCACA TGAGGAGACT GGGGCCTCCC ATACTCTCTA TGGTCATGGA

tFoxP2
+3 Val Cys Lys Trp Pro Gly Cys Glu Ser Val Cys Glu Asp Phe Gly Gln Phe Leu Lys His Leu Asn Asn Glu His Ala Leu Asp Asp Arg Ser Thr Ala Gln
2001 GTTTGCAAAT GGCCTGGCTG TGAAAGCGTT TGTGAAGATT TTGGACAATT TTTAAAGCAC CTTAACAATG AACATGCATT GGATGACAGA AGCACTGCTC

tFoxP2
+3 ·Glr Cys Arg Val Gln Met Gln Val Val Gln Gln Leu Glu Ile Gln Leu Ser Lys Glu Arg Glu Arg Leu Gln Ala Met Met Thr His Leu His Met Arg Pro·
2101 AGTGTCGGGT GCAAATGCAA GTGGTTCAGC AGTTAGAAAT ACAGCTTTCT AAAGAACGCG AACGTCTTCA AGCAATGATG ACCCACTTGC ACATGCGACC

tFoxP2 BamHI EcoRI
PsiI
+3 ·Pro Ser Glu Pro Lys Pro Ser Pro Lys Pro Val Ser Ala Tyr Cys Phe Ile Asn Ser Lys ***
2201 CTCGGAGCCC AAGCCATCTC CAAAACCTGT AAGTGCATAT TGCTTTATAA ACAGTAAATA GGTCTACGAA GGGCCTGTAC CTAGGATCCA GTGTGGTGGA
3'-T overhan[illegible]

PstI NotI XbaI
XhoI ApaI
EcoRI EcoRV AvaI PmeI
2301 ATTCTGCAGA TATCCAGCAC AGTGGCGGCC GCTCGAGTCT AGAGGGCCCG TTTAAACCCG CTGATCAGCC TCGACTGTGC CTTCTAGTTG CCAGCCATCT
2401 GTTGTTTGCC CCTCCCCCGT GCCTTCCTTG ACCCTGGAAG GTGCCACTCC CACTGTCCTT TCCTAATAAA ATGAGGAAAT TGCATCGCAT TGTCTGAGTA
2501 GGTGTCATTC TATTCTGGGG GGTGGGGTGG GGCAGGACAG CAAGGGGGAG GATTGGGAAG ACAATAGCAG GCATGCTGGG GATGCGGTGG GCTCTATGGC

PvuII
2601 TTCTGAGGCG GAAAGAACCA GCTGGGGCTC TAGGGGGTAT CCCCACGCGC CCTGTAGCGG CGCATTAAGC GCGGCGGGTG TGGTGGTTAC GCGCAGCGTG
2701 ACCGCTACAC TTGCCAGCGC CCTAGCGCCC GCTCCTTTCG CTTTCTTCCC TTCCTTTCTC GCCACGTTCG CCGGCTTTCC CCGTCAAGCT CTAAATCGGG
2801 GGCTCCCTTT AGGGTTCCGA TTTAGTGCTT TACGGCACCT CGACCCCAAA AAACTTGATT AGGGTGATGG TTCACGTAGT GGGCCATCGC CCTGATAGAC
2901 GGTTTTTCGC CCTTTGACGT TGGAGTCCAC GTTCTTTAAT AGTGGACTCT TGTTCCAAAC TGGAACAACA CTCAACCCTA TCTCGGTCTA TTCTTTTGAT

PsiI SV40 early promoter
3001 TTATAAGGGA TTTTGCCGAT TTCGGCCTAT TGGTTAAAAA ATGAGCTGAT TTAACAAAAA TTTAACGCGA ATTAATTCTG TGGAATGTGT GTCAGTTAGG

SV40 early promoter
3101 GTGTGGAAAG TCCCCAGGCT CCCCAGCAGG CAGAAGTATG CAAAGCATGC ATCTCAATTA GTCAGCAACC AGGTGTGGAA AGTCCCCAGG CTCCCCAGCA

SV40 early promoter
3201 GGCAGAAGTA TGCAAAGCAT GCATCTCAAT TAGTCAGCAA CCATAGTCCC GCCCCTAACT CCGCCCATCC CGCCCCTAAC TCCGCCCAGT TCCGCCCATT

SV40 early promoter
NcoI
3301 CTCCGCCCCA TGGCTGACTA ATTTTTTTTA TTTATGCAGA GGCCGAGGCC GCCTCTGCCT CTGAGCTATT CCAGAAGTAG TGAGGAGGCT TTTTTGGAGG

SV40 early promoter
SmaI
XmaI
AvaI

3401 CCTAGGCTTT TGCAAAAAGC TCCCGGGAGC TTGTATATCC ATTTTCGGAT CTGATCAGCA CGTGTTGACA ATTAATCATC GGCATAGTAT ATCGGCATAG

Zeo(R)
MscI
NcoI

3501 TATAATACGA CAAGGTGAGG AACTAAACCA TGGCCAAGTT GACCAGTGCC GTTCCGGTGC TCACCGCGCG CGACGTCGCC GGAGCGGTCG AGTTCTGGAC

Zeo(R)
SmaI
XmaI
AvaI AvaI

3601 CGACCGGCTC GGGTTCTCCC GGGACTTCGT GGAGGACGAC TTCGCCGGTG TGGTCCGGGA CGACGTGACC CTGTTCATCA GCGCGGTCCA GGACCAGGTG

Zeo(R)

3701 GTGCCGGACA ACACCCTGGC CTGGGTGTGG GTGCGCGGCC TGGACGAGCT GTACGCCGAG TGGTCGGAGG TCGTGTCCAC GAACTTCCGG GACGCCTCCG

Zeo(R)
ApaLI

3801 GGCCGGCCAT GACCGAGATC GGCGAGCAGC CGTGGGGGCG GGAGTTCGCC CTGCGCGACC CGGCCGGCAA CTGCGTGCAC TTCGTGGCCG AGGAGCAGGA

Zeo(R)

3901 CTGACACGTG CTACGAGATT TCGATTCCAC CGCCGCCTTC TATGAAAGGT TGGGCTTCGG AATCGTTTTC CGGGACGCCG GCTGGATGAT CCTCCAGCGC

PsiI

4001 GGGGATCTCA TGCTGGAGTT CTTCGCCCAC CCCAACTTGT TTATTGCAGC TTATAATGGT TACAAATAAA GCAATAGCAT CACAAATTTC ACAAATAAAG

SalI

4101 CATTTTTTTC ACTGCATTCT AGTTGTGGTT TGTCCAAACT CATCAATGTA TCTTATCATG TCTGTATACC GTCGACCTCT AGCTAGAGCT TGGCGTAATC

4201 ATGGTCATAG CTGTTTCCTG TGTGAAATTG TTATCCGCTC ACAATTCCAC ACAACATACG AGCCGGAAGC ATAAAGTGTA AAGCCTGGGG TGCCTAATGA

PvuII

4301 GTGAGCTAAC TCACATTAAT TGCGTTGCGC TCACTGCCCG CTTTCCAGTC GGGAAACCTG TCGTGCCAGC TGCATTAATG AATCGGCCAA CGCGCGGGGA

4401 GAGGCGGTTT GCGTATTGGG CGCTCTTCCG CTTCCTCGCT CACTGACTCG CTGCGCTCGG TCGTTCGGCT GCGGCGAGCG GTATCAGCTC ACTCAAAGGC

4501 GGTAATACGG TTATCCACAG AATCAGGGGA TAACGCAGGA AAGAACATGT GAGCAAAAGG CCAGCAAAAG GCCAGGAACC GTAAAAAGGC CGCGTTGCTG

4601 GCGTTTTTCC ATAGGCTCCG CCCCCCTGAC GAGCATCACA AAAATCGACG CTCAAGTCAG AGGTGGCGAA ACCCGACAGG ACTATAAAGA TACCAGGCGT

4701 TTCCCCCTGG AAGCTCCCTC GTGCGCTCTC CTGTTCCGAC CCTGCCGCTT ACCGGATACC TGTCCGCCTT TCTCCCTTCG GGAAGCGTGG CGCTTTCTCA

ApaLI

4801 TAGCTCACGC TGTAGGTATC TCAGTTCGGT GTAGGTCGTT CGCTCCAAGC TGGGCTGTGT GCACGAACCC CCCGTTCAGC CCGACCGCTG CGCCTTATCC

4901 GGTAACTATC GTCTTGAGTC CAACCCGGTA AGACACGACT TATCGCCACT GGCAGCAGCC ACTGGTAACA GGATTAGCAG AGCGAGGTAT GTAGGCGGTG

5001 CTACAGAGTT CTTGAAGTGG TGGCCTAACT ACGGCTACAC TAGAAGAACA GTATTTGGTA TCTGCGCTCT GCTGAAGCCA GTTACCTTCG GAAAAAGAGT

5101 TGGTAGCTCT TGATCCGGCA AACAAACCAC CGCTGGTAGC GGTTTTTTTG TTTGCAAGCA GCAGATTACG CGCAGAAAAA AAGGATCTCA AGAAGATCCT

5201 TTGATCTTTT CTACGGGGTC TGACGCTCAG TGGAACGAAA ACTCACGTTA AGGGATTTTG GTCATGAGAT TATCAAAAAG GATCTTCACC TAGATCCTTT

5301 TAAATTAAAA ATGAAGTTTT AAATCAATCT AAAGTATATA TGAGTAAACT TGGTCTGACA GTTACCAATG CTTAATCAGT GAGGCACCTA TCTCAGCGAT

Amp(R)

5401 CTGTCTATTT CGTTCATCCA TAGTTGCCTG ACTCCCCGTC GTGTAGATAA CTACGATACG GGAGGGCTTA CCATCTGGCC CCAGTGCTGC AATGATACCG

Amp(R)

5501 CGAGACCCAC GCTCACCGGC TCCAGATTTA TCAGCAATAA ACCAGCCAGC CGGAAGGGCC GAGCGCAGAA GTGGTCCTGC AACTTTATCC GCCTCCATCC

Amp(R)

5601 AGTCTATTAA TTGTTGCCGG GAAGCTAGAG TAAGTAGTTC GCCAGTTAAT AGTTTGCGCA ACGTTGTTGC CATTGCTACA GGCATCGTGG TGTCACGCTC

Amp(R)

5701 GTCGTTTGGT ATGGCTTCAT TCAGCTCCGG TTCCCAACGA TCAAGGCGAG TTACATGATC CCCCATGTTG TGCAAAAAAG CGGTTAGCTC CTTCGGTCCT

Amp(R)

PvuI

5801 CCGATCGTTG TCAGAAGTAA GTTGGCCGCA GTGTTATCAC TCATGGTTAT GGCAGCACTG CATAATTCTC TTACTGTCAT GCCATCCGTA AGATGCTTTT

Amp(R)

ScaI

5901 CTGTGACTGG TGAGTACTCA ACCAAGTCAT TCTGAGAATA GTGTATGCGG CGACCGAGTT GCTCTTGCCC GGCGTCAATA CGGGATAATA CCGCGCCACA

Amp(R)

6001 TAGCAGAACT TTAAAAGTGC TCATCATTGG AAAACGTTCT TCGGGGCGAA AACTCTCAAG GATCTTACCG CTGTTGAGAT CCAGTTCGAT GTAACCCACT

Amp(R)

ApaLI

6101 CGTGCACCCA ACTGATCTTC AGCATCTTTT ACTTTCACCA GCGTTTCTGG GTGAGCAAAA ACAGGAAGGC AAAATGCCGC AAAAAAGGGA ATAAGGGCGA

Amp(R)

6201 CACGGAAATG TTGAATACTC ATACTCTTCC TTTTTCAATA TTATTGAAGC ATTTATCAGG GTTATTGTCT CATGAGCGGA TACATATTTG AATGTATTTA

Amp(R)

SalI ApaLI

6301 GAAAAATAAA CAAATAGGGG TTCCGCGCAC ATTTCCCCGA AAAGTGCCAC CTGACGTCGA CGGATCGGGA GATCTCCCGA TCCCCTATGG TGCACTCTCA

6401 GTACAATCTG CTCTGATGCC GCATAGTTAA GCCAGTATCT GCTCCCTGCT TGTGTGTTGG AGGTCGCTGA GTAGTGCGCG AGCAAAATTT AAGCTACAAC

6501 AAGGCAAGGC TTGACCGACA ATTGCATGAA GAATCTGCTT AGGGTTAGGC GTTTTGCGCT GCTTCGCG

```
5′   gacgcgccctgtagcggcgcattaagcgcggcgggtgtggtggttacgcgcagcgtgaccgctac   65
3′   ctgcgcgggacatcgccgcgtaattcgcgccgcccacaccaccaatgcgcgtcgcactggcgatg

     acttgccagcgccctagcgcccgctcctttcgctttcttcccttcctttctcgccacgttcgccg   130
     tgaacggtcgcgggatcgcgggcgaggaaagcgaaagaagggaaggaaagagcggtgcaagcggc

     gctttccccgtcaagctctaaatcgggggctccctttagggttccgatttagtgctttacggcac   195
     cgaaaggggcagttcgagatttagcccccgagggaaatcccaaggctaaatcacgaaatgccgtg

     ctcgaccccaaaaaacttgattagggtgatggttcacgtagtgggccatcgccctgatagacggt   260
     gagctggggttttttgaactaatcccactaccaagtgcatcacccggtagcgggactatctgcca

     ttttcgccctttgacgttggagtccacgttctttaatagtggactcttgttccaaactggaacaa   325
     aaaagcgggaaactgcaacctcaggtgcaagaaattatcacctgagaacaaggtttgaccttgtt

     cactcaaccctatctcggtctattcttttgatttataagggattttgccgatttcggcctattgg   390
     gtgagttgggatagagccagataagaaaactaaatattccctaaaacggctaaagccggataacc

     ttaaaaaatgagctgatttaacaaaaatttaacgcgaattttaacaaaatattaacgtttacaat   455
     aatttttactcgactaaattgtttttaaattgcgcttaaaattgttttataattgcaaatgtta

     ttcaggtggcacttttcggggaaatgtgcgcggaacccctatttgtttatttttctaaatacatt   520
     aagtccaccgtgaaaagcccctttacacgcgccttggggataaacaaataaaaagatttatgtaa

     caaatatgtatccgctcatgagacaataaccctgataaatgcttcaataatattgaaaaaggaag   585
     gtttatacataggcgagtactctgttattgggactatttacgaagttattataactttttccttc

     agtatgagtattcaacatttccgtgtcgcccttattcccttttttgcggcattttgccttcctgt   650
     tcatactcataagttgtaaaggcacagcgggaataagggaaaaaacgccgtaaaacggaaggaca
```

1 Met Ser Ile Gln 5 His Phe Arg Val Ala 10 Leu Ile Pro Phe Phe 15 Ala Ala Phe Cys Leu 20 Pro Val

AMP

```
     ttttgctcacccagaaacgctggtgaaagtaaaagatgctgaagatcagttgggtgcacgagtgg   715
     aaaacgagtgggtctttgcgaccactttcattttctacgacttctagtcaacccacgtgctcacc
```

Phe Ala His 25 Pro Glu Thr Leu Val 30 Lys Val Lys Asp Ala 35 Glu Asp Gln Leu Gly 40 Ala Arg Val

AMP

gttacatcgaactggatctcaacagcggtaagatccttgagagttttcgccccgaagaacgtttt 780
caatgtagcttgacctagagttgtcgccattctaggaactctcaaaagcggggcttcttgcaaaa
45 50 55 60
Gly Tyr Ile Glu Leu Asp Leu Asn Ser Gly Lys Ile Leu Glu Ser Phe Arg Pro Glu Glu Arg Phe
AMP

ccaatgatgagcacttttaaagttctgctatgtggcgcggtattatcccgtattgacgccgggca 845
ggttactactcgtgaaaatttcaagacgatacaccgcgccataatagggcataactgcggcccgt
65 70 75 80 85
Pro Met Met Ser Thr Phe Lys Val Leu Leu Cys Gly Ala Val Leu Ser Arg Ile Asp Ala Gly Gln
AMP

agagcaactcggtcgccgcatacactattctcagaatgacttggttgagtactcaccagtcacag 910
tctcgttgagccagcggcgtatgtgataagagtcttactgaaccaactcatgagtggtcagtgtc
90 95 100 105
Glu Gln Leu Gly Arg Arg Ile His Tyr Ser Gln Asn Asp Leu Val Glu Tyr Ser Pro Val Thr
AMP

aaaagcatcttacggatggcatgacagtaagagaattatgcagtgctgccataaccatgagtgat 975
ttttcgtagaatgcctaccgtactgtcattctcttaatacgtcacgacggtattggtactcacta
110 115 120 125
Glu Lys His Leu Thr Asp Gly Met Thr Val Arg Glu Leu Cys Ser Ala Ala Ile Thr Met Ser Asp
AMP

PvuI
aacactgcggccaacttacttctgacaacgatcggaggaccgaaggagctaaccgcttttttgca 1040
ttgtgacgccggttgaatgaagactgttgctagcctcctggcttcctcgattggcgaaaaaacgt
130 135 140 145 150
Asn Thr Ala Ala Asn Leu Leu Leu Thr Thr Ile Gly Gly Pro Lys Glu Leu Thr Ala Phe Leu His
AMP

caacatgggggatcatgtaactcgccttgatcgttgggaaccggagctgaatgaagccataccaa 1105
gttgtaccccctagtacattgagcggaactagcaacccttggcctcgacttacttcggtatggtt
155 160 165 170
Asn Met Gly Asp His Val Thr Arg Leu Asp Arg Trp Glu Pro Glu Leu Asn Glu Ala Ile Pro
AMP

FspI
acgacgagcgtgacaccacgatgcctgtagcaatggcaacaacgttgcgcaaactattaactggc 1170
tgctgctcgcactgtggtgctacggacatcgttaccgttgttgcaacgcgtttgataattgaccg
175 180 185 190
Asn Asp Glu Arg Asp Thr Thr Met Pro Val Ala Met Ala Thr Thr Leu Arg Lys Leu Leu Thr Gly
AMP

```
gaactacttactctagcttcccggcaacaattaatagactggatggaggcggataaagttgcagg    1235
cttgatgaatgagatcgaagggccgttgttaattatctgacctacctccgcctatttcaacgtcc
195                 200                 205                 210                 215
Glu Leu Leu Thr Leu Ala Ser Arg Gln Gln Leu Ile Asp Trp Met Glu Ala Asp Lys Val Ala Gly
AMP >

accacttctgcgctcggcccttccggctggctggtttattgctgataaatctggagccggtgagc    1300
tggtgaagacgcgagccgggaaggccgaccgaccaaataacgactatttagacctcggccactcg
            220                 225                 230                 235
Pro Leu Leu Arg Ser Ala Leu Pro Ala Gly Trp Phe Ile Ala Asp Lys Ser Gly Ala Gly Glu
AMP >

gtgggtctcgcggtatcattgcagcactggggccagatggtaagccctcccgtatcgtagttatc    1365
cacccagagcgccatagtaacgtcgtgaccccggtctaccattcgggagggcatagcatcaatag
        240                 245                 250                 255
Arg Gly Ser Arg Gly Ile Ile Ala Ala Leu Gly Pro Asp Gly Lys Pro Ser Arg Ile Val Val Ile
AMP >

tacacgacggggagtcaggcaactatggatgaacgaaatagacagatcgctgagataggtgcctc    1430
atgtgctgcccctcagtccgttgatacctacttgctttatctgtctagcgactctatccacggag
260                 265                 270                 275                 280
Tyr Thr Thr Gly Ser Gln Ala Thr Met Asp Glu Arg Asn Arg Gln Ile Ala Glu Ile Gly Ala Ser
AMP >

actgattaagcattggtaactgtcagaccaagtttactcatatatactttagattgatttaaaac    1495
tgactaattcgtaaccattgacagtctggttcaaatgagtatatatgaaatctaactaaattttg
            285
Leu Ile Lys His Trp *
AMP

ttcattttaatttaaaaggatctaggtgaagatcctttttgataatctcatgaccaaaatccct    1560
aagtaaaaattaaattttcctagatccacttctaggaaaaactattagagtactggttttaggga

taacgtgagttttcgttccactgagcgtcagaccccgtagaaaagatcaaaggatcttcttgaga    1625
attgcactcaaaagcaaggtgactcgcagtctggggcatcttttctagtttcctagaagaactct

tcctttttttctgcgcgtaatctgctgcttgcaaacaaaaaaaccaccgctaccagcggtggttt    1690
aggaaaaaaagacgcgcattagacgacgaacgtttgtttttttggtggcgatggtcgccaccaaa

gtttgccggatcaagagctaccaactctttttccgaaggtaactggcttcagcagagcgcagata    1755
caaacggcctagttctcgatggttgagaaaaaggcttccattgaccgaagtcgtctcgcgtctat

ccaaatactgtccttctagtgtagccgtagttaggccaccacttcaagaactctgtagcaccgcc    1820
ggtttatgacaggaagatcacatcggcatcaatccggtggtgaagttcttgagacatcgtggcgg
```

```
tacatacctcgctctgctaatcctgttaccagtggctgctgccagtggcgataagtcgtgtctta
                                                                     1885
atgtatggagcgagacgattaggacaatggtcaccgacgacggtcaccgctattcagcacagaat

ccgggttggactcaagacgatagttaccggataaggcgcagcggtcgggctgaacggggggttcg
                                                                     1950
ggcccaacctgagttctgctatcaatggcctattccgcgtcgccagcccgacttgccccccaagc

tgcacacagcccagcttggagcgaacgacctacaccgaactgagatacctacagcgtgagcattg
                                                                     2015
acgtgtgtcgggtcgaacctcgcttgctggatgtggcttgactctatggatgtcgcactcgtaac

agaaagcgccacgcttcccgaagggagaaaggcggacaggtatccggtaagcggcagggtcggaa
                                                                     2080
tctttcgcggtgcgaagggcttccctctttccgcctgtccataggccattcgccgtcccagcctt

caggagagcgcacgagggagcttccagggggaaacgcctggtatctttatagtcctgtcgggttt
                                                                     2145
gtcctctcgcgtgctccctcgaaggtccccctttgcggaccatagaaatatcaggacagcccaaa

cgccacctctgacttgagcgtcgatttttgtgatgctcgtcaggggggcggagcctatggaaaaa
                                                                     2210
gcggtggagactgaactcgcagctaaaaacactacgagcagtccccccgcctcggataccttttt

cgccagcaacgcggcctttttacggttcctggccttttgctggccttttgctcacatgttctttc
                                                                     2275
gcggtcgttgcgccggaaaaatgccaaggaccggaaaacgaccggaaaacgagtgtacaagaaag

ctgcgttatcccctgattctgtggataaccgtattaccgcctttgagtgagctgataccgctcgc
                                                                     2340
gacgcaataggggactaagacacctattggcataatggcggaaactcactcgactatggcgagcg

cgcagccgaacgaccgagcgcagcgagtcagtgagcgaggaagcggaagagcgcctgatgcggta
                                                                     2405
gcgtcggcttgctggctcgcgtcgctcagtcactcgctccttcgccttctcgcggactacgccat

ttttctccttacgcatctgtgcggtatttcacaccgcagaccagccgcgtaacctggcaaaatcg
                                                                     2470
aaaagaggaatgcgtagacacgccataaagtgtggcgtctggtcggcgcattggaccgttttagc

gttacggttgagtaataaatggatgccctgcgtaagcgggtgtgggcggacaataaagtcttaaa
                                                                     2535
caatgccaactcattatttacctacgggacgcattcgcccacacccgcctgttatttcagaattt
                                                   Tn7L

ctgaacaaaatagatctaaactatgacaataaagtcttaaactagacagaatagttgtaaactga
                                                                     2600
gacttgttttatctagatttgatactgttatttcagaatttgatctgtcttatcaacatttgact
                                  Tn7L
```

```
aatcagtccagttatgctgtgaaaaagcatactggacttttgttatggctaaagcaaactcttca   2665
ttagtcaggtcaatacgacactttttcgtatgacctgaaaacaataccgatttcgtttgagaagt
Tn7L

                                                         BstXI
ttttctgaagtgcaaattgcccgtcgtattaaagaggggcgtggccaagggcatggtaaagacta   2730
aaaagacttcacgtttaacgggcagcataatttctccccgcaccggttcccgtaccatttctgat
Tn7L

tattcgcggcgttgtgacaatttaccgaacaactccgcggccgggaagccgatctcggcttgaac   2795
ataagcgccgcaacactgttaaatggcttgttgaggcgccggcccttcggctagagccgaacttg
Tn7L

gaattgttaggtggcggtacttgggtcgatatcaaagtgcatcacttcttcccgtatgcccaact   2860
cttaacaatccaccgccatgaacccagctatagtttcacgtagtgaagaagggcatacgggttga
        175                170                165                160
* Thr Ala Thr Ser Pro Asp Ile Asp Phe His Met Val Glu Glu Arg Ile Gly Leu Lys
Gentamicin

ttgtatagagagccactgcgggatcgtcaccgtaatctgcttgcacgtagatcacataagcacca   2925
aacatatctctcggtgacgccctagcagtggcattagacgaacgtgcatctagtgtattcgtggt
        155                150                145                140
Thr Tyr Leu Ala Val Ala Pro Asp Asp Gly Tyr Asp Ala Gln Val Tyr Ile Val Tyr Ala Gly
< Gentamicin

agcgcgttggcctcatgcttgaggagattgatgagcgcggtggcaatgccctgcctccggtgctc   2990
tcgcgcaaccggagtacgaactcctctaactactcgcgccaccgttacgggacggaggccacgag
        135                130                125                120
Leu Ala Asn Ala Glu His Lys Leu Leu Asn Ile Leu Ala Thr Ala Ile Gly Gln Arg Arg His Glu
< Gentamicin

gccggagactgcgagatcatagatatagatctcactacgcggctgctcaaacctgggcagaacgt   3055
cggcctctgacgctctagtatctatatctagagtgatgcgccgacgagtttggacccgtcttgca
115                110                105                100                95
Gly Ser Val Ala Leu Asp Tyr Ile Tyr Ile Glu Ser Arg Pro Gln Glu Phe Arg Pro Leu Val Tyr
< Gentamicin

aagccgcgagagcgccaacaaccgcttcttggtcgaaggcagcaagcgcgatgaatgtcttacta   3120
ttcggcgctctcgcggttgttggcgaagaaccagcttccgtcgttcgcgctacttacagaatgat
        90                85                80                75
Ala Ala Leu Ala Gly Val Val Ala Glu Gln Asp Phe Ala Ala Leu Ala Ile Phe Thr Lys Ser
< Gentamicin
```

BsmBI

```
cggagcaagttcccgaggtaatcggagtccggctgatgttgggagtaggtggctacgtctccgaa    3185
gcctcgttcaagggctccattagcctcaggccgactacaaccctcatccaccgatgcagaggctt
```

70 65 60 55
Arg Leu Leu Asn Gly Leu Tyr Asp Ser Asp Pro Gln His Gln Ser Tyr Thr Ala Val Asp Gly Phe

Gentamicin

PflFI
Tth111I

```
ctcacgaccgaaaagatcaagagcagcccgcatggatttgacttggtcagggccgagcctacatg    3250
gagtgctggcttttctagttctcgtcgggcgtacctaaactgaaccagtcccggctcggatgtac
```

50 45 40 35 30
Glu Arg Gly Phe Leu Asp Leu Ala Ala Arg Met Ser Lys Val Gln Asp Pro Gly Leu Arg Cys Thr

Gentamicin

```
tgcgaatgatgcccatacttgagccacctaactttgttttagggcgactgccctgctgcgtaaca    3315
acgcttactacgggtatgaactcggtggattgaaacaaaatcccgctgacgggacgacgcattgt
```

25 20 15 10
Arg Ile Ile Gly Met Ser Ser Gly Gly Leu Lys Thr Lys Pro Arg Ser Gly Gln Gln Thr Val

Gentamicin

```
tcgttgctgctgcgtaacatcgttgctgctccataacatcaaacatcgacccacggcgtaacgcg    3380
agcaacgacgacgcattgtagcaacgacgaggtattgtagtttgtagctgggtgccgcattgcgc
```

5 1
Asp Asn Ser Ser Arg Leu Met

Gentamicin

BsrGI

```
cttgctgcttggatgcccgaggcatagactgtacaaaaaaacagtcataacaagccatgaaaacc    3445
gaacgacgaacctacgggctccgtatctgacatgtttttttgtcagtattgttcggtacttttgg

gccactgcgccgttaccaccgctgcgttcggtcaaggttctggaccagttgcgtgagcgcatacg    3510
cggtgacgcggcaatggtggcgacgcaagccagttccaagacctggtcaacgcactcgcgtatgc

ctacttgcattacagtttacgaaccgaacaggcttatgtcaactgggttcgtgccttcatccgtt    3575
gatgaacgtaatgtcaaatgcttggcttgtccgaatacagttgacccaagcacggaagtaggcaa

tccacggtgtgcgtcacccggcaaccttgggcagcagcgaagtcgaggcatttctgtcctggctg    3640
aggtgccacacgcagtgggccgttggaacccgtcgtcgcttcagctccgtaaagacaggaccgac

gcgaacgagcgcaaggtttcggtctccacgcatcgtcaggcattggcggccttgctgttcttcta    3705
cgcttgctcgcgttccaaagccagaggtgcgtagcagtccgtaaccgccggaacgacaagaagat
```

cggcaaggtgctgtgcacggatctgccctggcttcaggagatcggaagacctcggccgtcgcggc 3770
gccgttccacgacacgtgcctagacgggaccgaagtcctctagccttctggagccggcagcgccg

gcttgccggtggtgctgaccccggatgaagtggttcgcatcctcggtttctggaaggcgagcat 3835
cgaacggccaccacgactggggcctacttcaccaagcgtaggagccaaagaccttccgctcgta

cgtttgttcgcccaggactctagctatagttctagtggttggctacattattgaagcatttatca 3900
gcaaacaagcgggtcctgagatcgatatcaagatcaccaaccgatgtaataacttcgtaaatagt

SphI
gggttattgtctcagagcatgcctgcaggcagctgcgcgctcgctcgctcactgaggccgcccgg 3965
cccaataacagagtctcgtacggacgtccgtcgacgcgcgagcgagcgagtgactccggcgggcc
AAV ITR

gcgtcgggcgacctttggtcgcccggcctcagtgagcgagcgagcgcgcagagagggagtggcca 4030
cgcagcccgctggaaaccagcgggccggagtcactcgctcgctcgcgcgtctctccctcaccggt
AAV ITR

MluI **SpeI**
actccatcactaggggttcctgcggccgcacgcgtgttactagttattaatagtaatcaattacg 4095
tgaggtagtgatccccaaggacgccggcgtgcgcacaatgatcaataattatcattagttaatgc
AAV ITR

gggtcattagttcatagcccatatatggagttccgcgttacataacttacggtaaatggcccgcc 4160
cccagtaatcaagtatcgggtatatacctcaaggcgcaatgtattgaatgccatttaccgggcgg

tggctgaccgcccaacgacccccgcccattgacgtcaataatgacgtatgttcccatagtaacgc 4225
accgactggcgggttgctgggggcgggtaactgcagttattactgcatacaagggtatcattgcg
CAG

caatagggactttccattgacgtcaatgggtggagtatttacggtaaactgcccacttggcagta 4290
gttatccctgaaaggtaactgcagttacccacctcataaatgccatttgacgggtgaaccgtcat
CAG

NdeI
catcaagtgtatcatatgccaagtacgccccctattgacgtcaatgacggtaaatggcccgcctg 4355
gtagttcacatagtatacggttcatgcgggggataactgcagttactgccatttaccgggcggac
CAG

```
                                                        SnaBI
gcattatgcccagtacatgaccttatgggactttcctacttggcagtacatctacgtattagtca   4420
cgtaatacgggtcatgtactggaatacccctgaaaggatgaaccgtcatgtagatgcataatcagt
                              CAG

tcgctattaccatggtcgaggtgagccccacgttctgcttcactctccccatctcccccccctcc   4485
agcgataatggtaccagctccactcggggtgcaagacgaagtgagaggggtagaggggggggagg
                              CAG

                                                              BsgI
ccaccccaattttgtatttatttattttttaattattttgtgcagcgatgggggcgggggggg   4550
ggtggggttaaaacataaataaataaaaaattaataaaacacgtcgctacccccgccccccccc
                              CAG

gggggggcgcgcgccaggcggggcggggcggggcgaggggcggggcggggcgaggcggagaggtg   4615
cccccccgcgcgcggtccgccccgccccgccccgctccccgccccgccccgctccgcctctccac
                              CAG

cggcggcagccaatcagagcggcgcgctccgaaagtttccttttatggcgaggcggcggcggcgg   4680
gccgccgtcggttagtctcgccgcgcgaggctttcaaaggaaaataccgctccgccgccgccgcc
                              CAG

cggccctataaaaagcgaagcgcgcggcgggcgggagtcgctgcgacgctgccttcgccccgtgc   4745
gccgggatatttttcgcttcgcgcgccgcccgccctcagcgacgctgcgacggaagcggggcacg
                              CAG

cccgctccgccgccgcctcgcgccgcccgccccggctctgactgaccgcgttactcccacaggtg   4810
gggcgaggcggcggcggagcgcggcgggcggggccgagactgactggcgcaatgagggtgtccac
                              CAG

                                        AfeI
agcgggcgggacggcccttctcctccgggctgtaattagcgcttggtttaatgacggcttgtttc   4875
tcgcccgccctgccgggaagaggaggcccgacattaatcgcgaaccaaattactgccgaacaaag
                              CAG

                                  PspOMI   ApaI
ttttctgtggctgcgtgaaagccttgaggggctccgggagggccctttgtgcgggggggagcggct   4940
aaaagacaccgacgcactttcggaactccccgaggccctcccgggaaacacgcccccctcgccga
                              CAG
```

cggggctgtccgcgggggggacggctgccttcgggggggacggggcagggcggggttcggcttctg 5005
gccccgacaggcgcccccctgccgacggaagcccccccctgccccgtcccgccccaagccgaagac
CAG

XbaI
gcgtgtgaccggcggctctagagcctctgctaaccatgttcatgccttcttctttttcctacagc 5070
cgcacactggccgccgagatctcggagacgattggtacaagtacggaagaagaaaaaggatgtcg
CAG

BamHI
tcctgggcaacgtgctggttattgtgctgtctcatcattttggcaaagaattggatccgccacca 5135
aggacccgttgcacgaccaataacacgacagagtagtaaaaccgtttcttaacctaggcggtggt
CAG
dnFoxP2

tgatgcaggaatctgcgacagagacaataagcaacagttcaatgaatcaaaatggaatgagcact 5200
actacgtccttagacgctgtctctgttattcgttgtcaagttacttagttttaccttactcgtga
1 5 10 15 20
Met Met Gln Glu Ser Ala Thr Glu Thr Ile Ser Asn Ser Ser Met Asn Gln Asn Gly Met Ser Thr
dnFoxP2

ctaagcagccaattagatgctggcagcagagatggaagatcaagtggtgacacgagcactgaagt 5265
gattcgtcggttaatctacgaccgtcgtctctaccttctagttcaccactgtgctcgtgacttca
25 30 35 40
Leu Ser Ser Gln Leu Asp Ala Gly Ser Arg Asp Gly Arg Ser Ser Gly Asp Thr Ser Thr Glu Val
dnFoxP2

aagcacagtagaactgctgcatctgcaacaacagcaggcactccaggcagcaaggcagctgcttt 5330
ttcgtgtcatcttgacgacgtagacgttgttgtcgtccgtgaggtccgtcgttccgtcgacgaaa
45 50 55 60 65
Ser Thr Val Glu Leu Leu His Leu Gln Gln Gln Gln Ala Leu Gln Ala Ala Arg Gln Leu Leu
dnFoxP2

AarI
BfuAI
BspMI
Bsu36I
tacagcaacagacaagtggactgaaatctcctaagggcagtgagaaacagagaccgctgcaggtg 5395
atgtcgttgtctgttcacctgactttagaggattcccgtcactctttgtctctggcgacgtccac
70 75 80 85
Leu Gln Gln Gln Thr Ser Gly Leu Lys Ser Pro Lys Gly Ser Glu Lys Gln Arg Pro Leu Gln Val
dnFoxP2

1472-correct-G-to-A-PCR-R

BbvCI
Bpu10I

cctgtgtcagtggccatgatgactccccaggtgatcacccctcagcaaatgcagcagattctcca 5460
ggacacagtcaccggtactactgaggggtccactagtggggagtcgtttacgtcgtctaagaggt

90 95 100 105
Pro Val Ser Val Ala Met Met Thr Pro Gln Val Ile Thr Pro Gln Gln Met Gln Gln Ile Leu Gln
dnFoxP2
1472-correct-G-to-A-PCR-R
1473-correct-G-to-A-PCR-F
A->G in plasmid

gcagcaagtgttgtctccgcagcagcttcaagcacttctccagcagcagcaagcagttatgttgc 5525
cgtcgttcacaacagaggcgtcgtcgaagttcgtgaagaggtcgtcgtcgttcgtcaatacaacg

110 115 120 125 130
Gln Gln Val Leu Ser Pro Gln Gln Leu Gln Ala Leu Leu Gln Gln Gln Gln Ala Val Met Leu
dnFoxP2

agcagcaacaactacaagagttttacaagaaacagcaagagcagttacatcttcagcttttgcag 5590
tcgtcgttgttgatgttctcaaaatgttctttgtcgttctcgtcaatgtagaagtcgaaaacgtc

135 140 145 150
Gln Gln Gln Gln Leu Gln Glu Phe Tyr Lys Lys Gln Gln Glu Gln Leu His Leu Gln Leu Leu Gln
dnFoxP2

cagcagcagcaacagcagcagcaacagcaacaacagcagcaacaacagcaacaacagcagcagca 5655
gtcgtcgtcgttgtcgtcgtcgttgtcgttgttgtcgtcgttgttgtcgttgttgtcgtcgtcgt

155 160 165 170
Gln Gln Gln Gln Gln Gln Gln Gln Gln Gln Gln Gln Gln Gln Gln Gln Gln Gln Gln Gln Gln Gln
dnFoxP2

gcaacaacaacaacagcagcagcagcaacagcaacagcagcaacagcatccaggaaagcaagcaa 5720
cgttgttgttgttgtcgtcgtcgtcgttgtcgttgtcgtcgttgtcgtaggtcctttcgttcgtt

175 180 185 190 195
Gln Gln Gln Gln Gln Gln Gln Gln Gln Gln Gln Gln Gln Gln Gln His Pro Gly Lys Gln Ala
dnFoxP2

aagagcaacagcagcagcagcagcagcagttggcagcccagcagcttgtcttccagcagcagctc 5785
ttctcgttgtcgtcgtcgtcgtcgtcgtcaaccgtcgggtcgtcgaacagaaggtcgtcgtcgag

200 205 210 215
Lys Glu Gln Gln Gln Gln Gln Gln Gln Gln Leu Ala Ala Gln Gln Leu Val Phe Gln Gln Gln Leu
dnFoxP2

BstAPI

ctccagatgcaacaacttcagcagcagcaacatctgctgaaccttcagcgtcagggactcatttc 5850
gaggtctacgttgttgaagtcgtcgtcgttgtagacgacttggaagtcgcagtccctgagtaaag

220 225 230 235
Leu Gln Met Gln Gln Leu Gln Gln Gln Gln His Leu Leu Asn Leu Gln Arg Gln Gly Leu Ile Ser
dnFoxP2

AflII

catcccacctggccaatctgctcttcctgtccagtctctgccacaagctggcttaagtcctgctg 5915
gtagggtggaccggttagacgagaaggacaggtcagagacggtgttcgaccgaattcaggacgac

240 245 250 255 260
Ile Pro Pro Gly Gln Ser Ala Leu Pro Val Gln Ser Leu Pro Gln Ala Gly Leu Ser Pro Ala
dnFoxP2

agattcagcagttatggaaagaagtgactggagttcacagtatggaagacaatggcattaaacat 5980
tctaagtcgtcaataccttcttcactgacctcaagtgtcataccttctgttaccgtaatttgta

265 270 275 280
Glu Ile Gln Gln Leu Trp Lys Glu Val Thr Gly Val His Ser Met Glu Asp Asn Gly Ile Lys His
dnFoxP2

ggagggctagacctcactactaacaattcctcctctactacctcctccaccacttccaaagcatc 6045
cctcccgatctggagtgatgattgttaaggaggagatgatggaggaggtggtgaaggtttcgtag

285 290 295 300
Gly Gly Leu Asp Leu Thr Thr Asn Asn Ser Ser Ser Thr Thr Ser Ser Thr Thr Ser Lys Ala Ser
dnFoxP2

accaccaataactcatcattccatagtgaatggacagtcttcagttctaaatgcaaggcgagaca 6110
tggtggttattgagtagtaaggtatcacttacctgtcagaagtcaagatttacgttccgctctgt

305 310 315 320 325
Pro Pro Ile Thr His His Ser Ile Val Asn Gly Gln Ser Ser Val Leu Asn Ala Arg Arg Asp
dnFoxP2

gctcgtcacatgaggagactggggcctcccatactctctatggtcatggagtttgcaaatggcct 6175
cgagcagtgtactcctctgaccccggagggtatgagagataccagtacctcaaacgtttaccgga

330 335 340 345
Ser Ser Ser His Glu Glu Thr Gly Ala Ser His Thr Leu Tyr Gly His Gly Val Cys Lys Trp Pro
dnFoxP2

NsiI

ggctgtgaaagcgtttgtgaagattttggacaatttttaaagcaccttaacaatgaacatgcatt 6240
ccgacactttcgcaaacacttctaaaacctgttaaaaatttcgtggaattgttacttgtacgtaa

350 355 360 365
Gly Cys Glu Ser Val Cys Glu Asp Phe Gly Gln Phe Leu Lys His Leu Asn Asn Glu His Ala Leu
dnFoxP2

ggatgacagaagcactgctcagtgtcgggtgcaaatgcaagtggttcagcagttagaaatacagc 6305
cctactgtcttcgtgacgagtcacagcccacgtttacgttcaccaagtcgtcaatctttatgtcg
370 375 380 385 390
Asp Asp Arg Ser Thr Ala Gln Cys Arg Val Gln Met Gln Val Val Gln Gln Leu Glu Ile Gln
dnFoxP2

tttctaaagaacgcgaacgtcttcaagcaatgatgacccacttgcacatgcgaccctcggagccc 6370
aaagatttcttgcgcttgcagaagttcgttactactgggtgaacgtgtacgctgggagcctcggg
395 400 405 410
Leu Ser Lys Glu Arg Glu Arg Leu Gln Ala Met Met Thr His Leu His Met Arg Pro Ser Glu Pro
dnFoxP2

SalI AccI TliI XhoI PaeR7I
aagccatctccaaaacctgtaagtgcatattgctttataaacagtaaataggtcgacctcgagaa 6435
ttcggtagaggttttggacattcacgtataacgaaatatttgtcatttatccagctggagctctt
415 420 425
Lys Pro Ser Pro Lys Pro Val Ser Ala Tyr Cys Phe Ile Asn Ser Lys *
dnFoxP2
WPRE

tcaacctctggattacaaaatttgtgaaagattgactggtattcttaactatgttgctcctttta 6500
agttggagacctaatgttttaaacactttctaactgaccataagaattgatacaacgaggaaaat
WPRE

cgctatgtggatacgctgctttaatgcctttgtatcatgctattgcttcccgtatggctttcatt 6565
gcgatacacctatgcgacgaaattacggaaacatagtacgataacgaagggcataccgaaagtaa
WPRE

ttctcctccttgtataaatcctggttgctgtctctttatgaggagttgtggcccgttgtcaggca 6630
aagaggaggaacatatttaggaccaacgacagagaaatactcctcaacaccgggcaacagtccgt
WPRE

acgtggcgtggtgtgcactgtgtttgctgacgcaacccccactggttggggcattgccaccacct 6695
tgcaccgcaccacacgtgacacaaacgactgcgttgggggtgaccaaccccgtaacggtggtgga
WPRE

gtcagctcctttccgggactttcgctttccccctccctattgccacggcggaactcatcgccgcc 6760
cagtcgaggaaaggccctgaaagcgaaagggggagggataacggtgccgccttgagtagcggcgg
WPRE

tgccttgcccgctgctggacaggggctcggctgttgggcactgacaattccgtggtgttgtcggg 6825
acggaacgggcgacgacctgtccccgagccgacaacccgtgactgttaaggcaccacaacagccc
WPRE

gaagctgacgtcctttccatggctgctcgcctgtgttgccacctggattctgcgcgggacgtcct 6890
cttcgactgcaggaaaggtaccgacgagcggacacaacggtggacctaagacgcgccctgcagga
WPRE

tctgctacgtcccttcggccctcaatccagcggaccttccttcccgcggcctgctgccggctctg 6955
agacgatgcagggaagccgggagttaggtcgcctggaaggaagggcgccggacgacggccgagac
WPRE

cggcctcttccgcgtcttcgccttcgccctcagacgagtcggatctccctttgggccgcctcccc 7020
gccggagaaggcgcagaagcggaggcgggagtctgctcagcctagagggaaacccggcggagggg
WPRE

Acc65I KpnI
gcggtacccaattcgccctatagtgagtcgtattacgcgcgcagcggccgaccatggcccaactt 7085
cgccatgggttaagcgggatatcactcagcataatgcgcgcgtcgccggctggtaccgggttgaa
WPRE SV40pA-144bp

gtttattgcagcttataatggttacaaataaagcaatagcatcacaaatttcacaaataaagcat 7150
caaataacgtcgaatattaccaatgtttatttcgttatcgtagtgtttaaagtgtttatttcgta
SV40pA-144bp

tttttcactgcattctagttgtggtttgtccaaactcatcaatgtatcttatcatgtctggatc 7215
aaaaagtgacgtaagatcaacaccaaacaggtttgagtagttacatagaatagtacagacctag
SV40pA-144bp

BspEI PmlI RsrII
tccggaccacgtgcggaccgagcggccgcaggaacccctagtgatggagttggccactccctctc 7280
aggcctggtgcacgcctggctcgccggcgtccttggggatcactacctcaaccggtgagggagag
SV40pA-144bp AAV ITR

tgcgcgctcgctcgctcactgaggccgggcgaccaaaggtcgcccgacgcccgggctttgcccgg 7345
acgcgcgagcgagcgagtgactccggcccgctggtttccagcgggctgcgggcccgaaacgggcc
AAV ITR

```
                                                          HindIII
gcggcctcagtgagcgagcgagcgcgcagctgcctgcaggaagctgtaagcttgtcgagaagtac     7410
cgccggagtcactcgctcgctcgcgcgtcgacggacgtccttcgacattcgaacagctcttcatg
AAV ITR

tagaggatcataatcagccataccacatttgtagaggttttacttgctttaaaaaacctcccaca     7475
atctcctagtattagtcggtatggtgtaaacatctccaaaatgaacgaaattttttggagggtgt
SV40pA-237bp

                                         MfeI         HpaI
cctcccctgaacctgaaacataaaatgaatgcaattgttgttgttaacttgtttattgcagctt     7540
ggaggggacttggactttgtatttacttacgttaacaacaacaattgaacaaataacgtcgaa
SV40pA-237bp

ataatggttacaaataaagcaatagcatcacaaatttcacaaataaagcatttttttcactgcat     7605
tattaccaatgtttatttcgttatcgtagtgtttaaagtgtttatttcgtaaaaaaagtgacgta
SV40pA-237bp

tctagttgtggtttgtccaaactcatcaatgtatcttatcatgtctggatctgatcactgatatc     7670
agatcaacaccaaacaggtttgagtagttacatagaatagtacagacctagactagtgactatag
SV40pA-237bp

  AvrII
gcctaggagatccgaaccagataagtgaaatctagttccaaactattttgtcattttaatttc     7735
cggatcctctaggcttggtctattcactttagatcaaggtttgataaaacagtaaaaattaaaag
Tn7R

gtattagcttacgacgctacacccagttcccatctattttgtcactcttccctaaataatcctta     7800
cataatcgaatgctgcgatgtgggtcaagggtagataaaacagtgagaagggatttattaggaat
Tn7R

aaaactccatttccacccctcccagttcccaactatttgtccgcccacagcggggcatttttct     7865
ttttgaggtaaaggtggggagggtcaagggttgataaaacaggcgggtgtcgccccgtaaaaaga
Tn7R

tcctgttatgtttttaatcaaacatcctgccaactccatgtgacaaaccgtcatcttcggctact     7930
aggacaatacaaaaattagtttgtaggacggttgaggtacactgtttggcagtagaagccgatga
```

```
ttttctctgtcacagaatgaaaattttctgtcatctcttcgttattaatgtttgtaattgactg
                                                                      7995
aaaagagacagtgtcttacttttaaaagacagtagagaagcaataattacaaacattaactgac

aatatcaacgcttatttgcagcctgaatggcgaatg     3'
                                         ••• 8031
ttatagttgcgaataaacgtcggacttaccgcttac     5'
```
