# Supplementary material for: FoxP2 isoforms delineate spatiotemporal transcriptional networks for vocal learning in the zebra finch
Source: eLife. 2018 Jan 23;7:e30649. doi: 10.7554/eLife.30649 (PMC5826274; doi:10.7554/eLife.30649)
Supplement: Transparent reporting form [file elife-30649-transrepform.docx]

***eLife’s* transparent reporting form**

We encourage authors to provide detailed information *within their submission* to facilitate the interpretation and replication of experiments. If you have any questions, please contact us: [editorial@elifesciences.org](mailto:editorial@elifesciences.org).

**Sample-size estimation**

- You should state whether an appropriate sample size was computed when the study was being designed
- You should state the statistical method of sample size computation and any required assumptions
- If no explicit power analysis was used, you should describe how you decided what sample (replicate) size (number) to use

Please outline where this information can be found within the submission (e.g., page numbers or figure legends), or explain why this information doesn’t apply to your submission:

Sample size for animals used and rationale are presented on page 17, lines 457-462.

qRT-PCR was performed on total RNA from birds used in the RNA-seq experiment so that 19 samples were used. This information is presented in the figure legend for **Panel 1C:** Page 31, lines 842-850. Reactions were performed in triplicate, as denoted in Methods, page 19, lines 529-530.

The sample sizes for acoustic data feature analysis were determined by the number of unique and readily identified syllables sung by each bird. These data are presented in the figure legends, as follows:

**Panel 2C:** Page 31, lines 865-872.

**Figure 2-figure supplement 1:** Same as panel 2C, data are transformed

Acoustic similarity data were calculated between each bird and its tutor and presented in **Panel 2D**. The sample sizes are presented in the figure legend on page 31, lines 873-881.

Gene networks were constructed from expression data generated from all 19 samples. This is denoted on page 25, lines 643-645.

**Replicates**

- You should report how often each experiment was performed
- You should include a definition of biological versus technical replication
- The data obtained should be provided and sufficient information should be provided to indicate the number of independent biological and/or technical replicates
- If you encountered any outliers, you should describe how these were handled
- Criteria for exclusion/inclusion of data should be clearly stated
- High-throughput sequence data should be uploaded before submission, with a private link for reviewers provided (these are available from both GEO and ArrayExpress)

Please outline where this information can be found within the submission (e.g., page numbers or figure legends), or explain why this information doesn’t apply to your submission:

The experimental timeline begins on page 18, line 469.

Biological replicates are equal to the number of animals in each virus group, as described on page 19, line 485.

Technical replicates in the RNA-seq experiments were performed by sequencing each sample in two sequencing lanes, as denoted on page 24, lines 623-624. We checked for batch effects based on sequencing lane and found no effect, as described on page 24, line 638.

Our RNA-seq preprocessing steps removed outlier expression data, which are described on page 24, lines 626-645.

Raw and processed RNA-seq and expression data are available at GEO and referenced on page 33, lines 875-879: <https://www.ncbi.nlm.nih.gov/geo/query/acc.cgi?token=qfobwsychpwrnid&acc=GSE96843>

**Statistical reporting**

- Statistical analysis methods should be described and justified
- Raw data should be presented in figures whenever informative to do so (typically when N per group is less than 10)
- For each experiment, you should identify the statistical tests used, exact values of N, definitions of center, methods of multiple test correction, and dispersion and precision measures (e.g., mean, median, SD, SEM, confidence intervals; and, for the major substantive results, a measure of effect size (e.g., Pearson's r, Cohen's d)
- Report exact p-values wherever possible alongside the summary statistics and 95% confidence intervals. These should be reported for all key questions and not only when the p-value is less than 0.05.

**Panel 1C:** Statistical information is described in the figure legend on pages 34-35, lines 896-904.

**Panel 2C:** Mean and SEM for each feature where a difference was observed in one group are reported in the figure legend on page 35, lines 919-928. Result of one-way ANOVA is presented on page 36, lines 936-937. Non-significant results are not reported.

**Panel 2D:** Mean and SEM for percentage similarity attained on day of sacrifice are presented in the figure legend on page 35-36, lines 929-932. Only 65d mean and SEM are stated in the figure legend since they are the values correlated to gene expression. One-way ANOVA results for all bins are presented on page 36, lines 934-936.

**Panel 3B, Figure 3 Figure Supplements 1-3:** Pearson’s rho and p-values are denoted on the panel and described in the figure legend on page 36-37, lines 955-959. Description of p-value correction and rationale for modules selected in downstream analysis are in the “Correlation of behavior to gene expression” section, beginning on line 707.

**Panels 3C, 4D, 5D:** Pearson’s rho, p-values are denoted on the panel and the statistical test is named in the figure legends for these panels.

**Panels 4B, 5B:** Description of module preservation statistics calculations are described in Methods, beginning on line 675. Exhaustive module preservation statistics are presented in **Supplementary Files 3 and 4**.

Please outline where this information can be found within the submission (e.g., page numbers or figure legends), or explain why this information doesn’t apply to your submission:

(For large datasets, or papers with a very large number of statistical tests, you may upload a single table file with tests, Ns, etc., with reference to page numbers in the manuscript.)

**Additional data files (“source data”)**

- We encourage you to upload relevant additional data files, such as numerical data that are represented as a graph in a figure, or as a summary table
- Where provided, these should be in the most useful format, and they can be uploaded as “Source data” files linked to a main figure or table
- Include model definition files including the full list of parameters used
- Include code used for data analysis (e.g., R, MatLab)
- Avoid stating that data files are “available upon request”

Please indicate the figures or tables for which source data files have been provided:

We have provided source data for the following figure panels and are happy to make available any additional data:

Figure 1 – Source Data 1: Averaged Ct values for each triplicate qPCR reaction presented in **Panel 1C**.

Figure 2 – Source Data 1: Effect sizes for each syllable presented in **Panel 2C**.

Figure 2 – Source Data 2: The binned similarity scores presented in the upper portion of **Panel 2D**.

Figure 3 – Source Code 1: An R workspace containing the network presented in **Panel 3A**.

Figure 3 – Source Code 2: An R workspace containing the processed expression data for the network presented in **Panel 3A**.

Figure 3 – Source Data 3: The behavioral scores for each animal that were correlated to the Area X network, as presented in **Panels 3A and 3B**.

Figure 3 – Source Data 4: The Pearson correlation data for each module eigengene and the behavioral traits presented in Figure 3 - Source Data 3 and **Panel 3B**.

Figure 4 – Source Data 1: An R workspace containing the network presented in **Panel 4A**.

Figure 4 – Source Data 2: An R workspace containing the processed expression data for the network presented in **Panel 4A**.

Figure 5 – Source Data 1: An R workspace containing networks and expression data for adult and juvenile birds composed of genes that were present in the original versions of both networks. These data were used to generate all panels in **Figure 5**.

Figure 6 – Source Data 1: The sorted gene expression data as presented in **Panel 6A**. Columns are ordered from left to right by increasing tutor percentage similarity attained by that bird. Rows are sorted by the gene’s significance to learning. **Panel 6A** is a graphical representation of the top and bottom 20 rows from this table.

Figure 7 – Source Data 1: A .gexf edgelist containing the information presented in **Figure 7**. File is suitable for opening in Gephi plotting software, as described in Methods.

In addition, we provide supplementary files containing network and behavior summary data for all genes in juvenile Area X and VSP and adult Area X networks as Supplementary File 1. Gene ontology information for all genes and modules are presented in Supplementary File 2. Module preservation statistics for juvenile Area X vs. juvenile VSP (as described in Figure 4) are presented in Supplementary File 3. Module preservation statistics for juvenile Area X vs. adult Area X (as described in Figure 5) are presented in Supplementary File 4. All protein interaction data are presented in Supplementary File 5.

Our data are presented as interactive figures on a website created for the manuscript (<https://www.ibp.ucla.edu/research/white/genenetwork.html)>.
